# Cathepsin-D in primary breast cancer: prognostic evaluation involving 2810 patients

**DOI:** 10.1038/sj.bjc.6990048

**Published:** 1999-01

**Authors:** J A Foekens, M P Look, J Bolt-de Vries, M E Meijer-van Gelder, W L J van Putten, J G M Klijn

**Affiliations:** Division of Endocrine Oncology, Department of Medical Oncology, Academic Hospital Rotterdam, Rotterdam, The Netherlands; Department of Statistics, Rotterdam Cancer Institute (Daniel den Hoed Kliniek), Academic Hospital Rotterdam, Rotterdam, The Netherlands

**Keywords:** cathepsin-D, breast cancer, breast cancer prognosis, multivariate analysis

## Abstract

There is controversy regarding the prognostic value of cathepsin-D in primary breast cancer. An increased level of cathepsin-D in tumour extracts has been found to be associated with a poor relapse-free and overall survival. Studies performed with immunohistochemistry or Western blotting have produced diverse results. We have analysed 2810 cytosolic extracts obtained from human primary breast tumours for cathepsin-D expression, and have correlated their levels with prognosis. The median follow-up of the patients still alive was 88 months. Patients with high cathepsin-D levels had a significantly worse relapse-free and overall survival, also in multivariate analysis (*P* < 0.0001). Adjuvant therapy which was associated with an improved prognosis in node-positive patients in univariate analysis, also significantly added to the multivariate models for relapse-free and overall survival. There were no statistically significant interactions between the levels of cathepsin-D and any of the classical prognostic factors in analysis for relapse-free survival, suggesting that the prognostic value of cathepsin-D is not different in the various subgroups of patients. Indeed, multivariate analyses in subgroups of node-negative and -positive patients, pre- and post-menopausal patients, and their combinations, showed that tumours with high cathepsin-D values had a significantly poor relapse-free survival, with relative hazard rates ranging from 1.3 to 1.5, compared with tumours with low cathepsin-D levels. The results presented here on 2810 patients confirm that high cytosolic cathepsin-D values are associated with poor prognosis in human primary breast cancer. © 1999 Cancer Research Campaign


*British Journal of Cancer* (1999) **79**(2), 300–307
© 1999 Cancer Research Campaign

### Cathepsin-D in primary breast cancer: prognostic evaluation involving 2810 patients

**JA Foekens[1], MP Look[1], J Bolt-de Vries[1], ME Meijer-van Gelder[1], WLJ van Putten[2] and JGM Klijn[1]**

[1]Division of Endocrine Oncology, Department of Medical Oncology, and [2]Department of Statistics, Rotterdam Cancer Institute (Daniel den Hoed Kliniek), Academic Hospital Rotterdam, The Netherlands

**Summary** There is controversy regarding the prognostic value of cathepsin-D in primary breast cancer. An increased level of cathepsin-D in tumour extracts has been found to be associated with a poor relapse-free and overall survival. Studies performed with immunohistochemistry or Western blotting have produced diverse results. We have analysed 2810 cytosolic extracts obtained from human primary breast tumours for cathepsin-D expression, and have correlated their levels with prognosis. The median follow-up of the patients still alive was 88 months. Patients with high cathepsin-D levels had a significantly worse relapse-free and overall survival, also in multivariate analysis ($P < 0.0001$). Adjuvant therapy which was associated with an improved prognosis in node-positive patients in univariate analysis, also significantly added to the multivariate models for relapse-free and overall survival. There were no statistically significant interactions between the levels of cathepsin-D and any of the classical prognostic factors in analysis for relapse-free survival, suggesting that the prognostic value of cathepsin-D is not different in the various subgroups of patients. Indeed, multivariate analyses in subgroups of node-negative and -positive patients, pre- and post-menopausal patients, and their combinations, showed that tumours with high cathepsin-D values had a significantly poor relapse-free survival, with relative hazard rates ranging from 1.3 to 1.5, compared with tumours with low cathepsin-D levels. The results presented here on 2810 patients confirm that high cytosolic cathepsin-D values are associated with poor prognosis in human primary breast cancer.

**Keywords:** cathepsin-D; breast cancer; breast cancer prognosis; multivariate analysis

Cathepsin-D is a lysosomal aspartyl protease which is expressed in all tissues. In breast cancer, it was first identified as a 52-kDa oestrogen-regulated secretory glycoprotein with autocrine mitogenic activity (Westley and Rochefort, 1979, 1980; Vignon et al, 1986; Rochefort, 1994; Westley and May, 1996). In oestrogen receptor (ER)-positive breast cancer cells, its gene transcription is increased by oestrogen and growth factors, whereas in ER-negative breast cancer cells it is constitutively expressed by an unknown mechanism. Many biological roles have been attributed to cathepsin-D (reviewed by Westley and May, 1996), including, among others: degradation of the extracellular matrix (Briozzo et al, 1988), increasing cells' malignant phenotype and metastatic potential (Garcia et al, 1990), stimulation of (metastatic) cell proliferation by increasing the local bioavailability of growth-stimulatory growth factors (Briozzo et al, 1991; Conover and De Leon, 1994), inactivation of a growth inhibitor (Liaudet et al, 1995), and prevention of apoptosis (Saftig et al, 1995).

In patients with primary breast cancer, overexpression of cathepsin-D was found to be related to a poor prognosis (Thorpe et al, 1989; Spyratos et al, 1989), in analogy with observations made with the serine protease urokinase-type plasminogen activator (uPA), of which increased activity (Duffy et al, 1988) and antigen level (Jänicke et al, 1990) have been shown to be associated with a poor prognosis. The initial studies of Thorpe et al (1989) and Spyratos et al (1989), using quantitative immunoassays (enzyme-linked immunosorbent assay, immunoradiometric assay) to assess cytosolic cathepsin-D levels, have been confirmed by many others employing the same technique (reviewed by Rochefort, 1994; Westley and May, 1996). However, utilization of other methods to assess cathepsin-D status, i.e. Western blotting and immunohistochemistry, resulted in discrepant results (Henry et al, 1990; Tandon et al, 1990; Domagala et al, 1992, Isola et al, 1993; Ravdin, 1993, Ravdin et al, 1994; Westley and May, 1996). These conflicting results have been attributed to the use of different antibodies without standardized quantification (Cardiff, 1994; Rochefort, 1996, Westley and May, 1996), a problem which is not encountered when quantitative immunoassays on cytosolic extracts are used (Benraad et al, 1992).

Notwithstanding the drawbacks of immunohistochemistry and contrasting data (Cardiff, 1996; Emmert-Buck, 1996; Rochefort, 1996, Westley and May, 1996), there is evidence that the expression of cathepsin-D by host stromal cells (Têtu et al, 1993; Joensuu et al, 1995; O'Donoghue et al, 1995; Nadji et al, 1996), or the cancer cells (Isola et al, 1993), is associated with prognosis. It has been suggested that measuring total cathepsin-D levels in tumour extracts (comprising tumour cells and host cells) has no practical value (Nadji et al, 1996). This firm conclusion drawn from data obtained from only 154 patients is surprising. In contrast to conflicting data obtained with immunohistochemistry, there exists ample evidence for an independent relationship of poor prognosis with high cathepsin-D levels measured in breast tumour extracts (Rochefort, 1994; Westley and May, 1996). This is also true for the clinically most relevant subset of node-negative patients (Spyratos et al, 1989; Thorpe et al, 1989; Kute et al, 1992; Foekens et al, 1993, 1996; Westley and May, 1996; Ferrandina et al, 1997).

*Received 12 December 1997*
*Revised 16 April 1998*
*Accepted 29 April 1998*

*Correspondence to:* JA Foekens, Rotterdam Cancer Institute (Daniel den Hoed Kliniek), Groene Hilledijk 301, 3075 EA Rotterdam, The Netherlands

**Table 1** Relationship between cathepsin-D levels and patient and tumour characteristics

| Characteristic | Number of patients[a] | Percentage of patients according to cathepsin-D content (pmol $mg^{-1}$ protein) | | | | *P*-value |
|---|---|---|---|---|---|---|
| | | 0–33 | > 33–47 | > 47–70 | > 70 | |
| All patients | 2810 | 25 | 25 | 25 | 25 | |
| Age at surgery (years) | | | | | | 0.01[b] |
| ≤ 40 | 326 | 26 | 27 | 24 | 23 | |
| 40–55 | 1008 | 27 | 24 | 25 | 24 | |
| 56–70 | 960 | 23 | 26 | 26 | 25 | |
| > 70 | 516 | 25 | 23 | 24 | 28 | |
| Menopausal status | | | | | | 0.03[c] |
| Premenopausal | 1112 | 26 | 26 | 24 | 24 | |
| Post menopausal | 1698 | 24 | 24 | 25 | 26 | |
| Tumour size | | | | | | 0.0001[c] |
| T1 | 1198 | 29 | 24 | 24 | 23 | |
| T2 | 1345 | 21 | 26 | 27 | 27 | |
| T3/4 | 267 | 26 | 25 | 21 | 27 | |
| Nodal status | | | | | | 0.0001[c] |
| No | 1412 | 28 | 27 | 25 | 20 | |
| N1–3 | 708 | 23 | 25 | 26 | 26 | |
| N > 3 | 661 | 19 | 21 | 25 | 34 | |
| Grade | | | | | | 0.1[c] |
| Well/moderate | 576 | 27 | 25 | 23 | 25 | |
| Poor | 1561 | 23 | 25 | 26 | 26 | |
| ER positive[d] | | | | | | < 0.0001[b] |
| No | 604 | 28 | 27 | 24 | 21 | |
| Yes | 2130 | 24 | 24 | 25 | 27 | |
| PgR positive[d] | | | | | | < 0.0001[b] |
| No | 798 | 27 | 25 | 26 | 23 | |
| Yes | 1888 | 24 | 24 | 25 | 26 | |

[a]Because of missing values, the numbers do not always add up to 2810. [b]*P*-value for Spearman rank correlation. [c]*P*-value for Kruskall–Wallis test, including a Wilcoxon-type test for trend across ordered groups, when appropriate. [d]Cut-off point used for ER and PgR: 10 fmol $mg^{-1}$ protein.

In the present definitive study, we have determined cytosolic cathepsin-D values by IRMA in 2810 patients with primary breast cancer and have correlated these levels with patient and tumour characteristics and prognosis.

### PATIENTS AND METHODS

### Patients and tissues

Inclusion criteria for the 2810 patients from whom tumour or cytosol samples were stored in our tumour bank (liquid nitrogen) were: primary diagnosis of breast cancer between 1978 and 1992 (at least 5 years of potential follow-up); no metastatic disease at diagnosis; no previous diagnosis of carcinoma, with the exception of basal cell skin carcinoma and cervical cancer stage I; no evidence of disease within 1 month of primary surgery. In case of mastectomy after an initial lumpectomy because of residual disease, the mastectomy is considered as (part of) the primary treatment. Patients with inoperable T4 tumours and patients who received neoadjuvant treatment before primary surgery were excluded. Median age of the patients at the time of surgery (modified mastectomy, 1502 patients; breast conserving lumpectomy, 1308 patients) was 57 years (range 24–94 years). Radiotherapy was given to 76% of the patients: on the breast/thoracic wall in 1787 patients and/or on the axilla in 763 patients, and/or on one or more lymph node areas other than the axilla in 894 patients. None of the node-negative patients received adjuvant therapy. Of the 1369 node-positive patients, adjuvant chemotherapy (mainly CMF; cyclophosphamide, methotrexate, 5-fluorouracil) was given to 451 patients, whereas 206 patients received adjuvant hormonal therapy either alone (183 patients) or in combination with chemotherapy (23 patients). All patients were routinely examined every 3–6 months during the first 5 years of follow-up and once a year thereafter. Of the 2810 patients, 147 patients (5%) died without evidence of disease and were censored at last follow-up in the analysis for relapse-free survival. During follow-up, 1306 (46%) patients showed a relapse and were counted as failures in the analysis for relapse-free survival. Nine hundred and forty-two (34%) patients died after a previous relapse. A total of 1089 (147 + 942) were counted as failures in the analysis for overall survival. The median follow-up period of patients still alive was 88 months (range 1–207 months). Further characteristics of patients and tumours are listed in Table 1.

### Assay of cathepsin-D, oestrogen receptor (ER) and progesterone receptor (PgR)

Tumour tissues were stored in (liquid nitrogen) and pulverized in the frozen state with a microdismembrator following the recommendations of the European Organization for Research and Treatment of Cancer (EORTC) for processing of breast tumour tissue for cytosolic ER and PgR determinations (EORTC Breast

© Cancer Research Campaign 1999

Cancer Cooperative Group, 1980). The resulting tissue powder was suspended in EORTC receptor buffer (10 mM dipotassium hydrogen phosphate buffer, containing 1.5 mM dipotassium chloride EDTA, 3 mM sodium azide, 10 mM monothioglycerol and 10% v/v glycerol, pH 7.4). The suspension was centrifuged for 30 min at 100 000 × ***g*** to obtain the supernatant fraction (cytosol). ER and PgR levels were determined by ligand binding assay or enzyme immunoassay as described before (Foekens et al, 1989). The cut-off level used to classify tumours as ER or PgR positive and negative was 10 fmol $mg^{-1}$ cytosolic protein.

Cathepsin-D levels were determined in breast tumour cytosols with a radiometric immunoassay (ELSA-CATH-D; CIS bio international, Gif-sur-Yvette, France). To enable the assessment of the between-assay variations (%CV), in each assay run an aliquot of a pooled breast cancer cytosol sample was analysed. Over a period of 7 years, the between-assay coefficient of variation (CV) was 8.2%. The within-assay CVs of samples measured in duplicate was 2.9%.

### Statistical analysis

The associations of cathepsin-D with other variables were tested with non-parametric tests: with Spearman rank correlation ($r_s$) for continuous variables (age, ER, PgR), and the Wilcoxon rank-sum test or Kruskall–Wallis test, including a Wilcoxon-type test for trend across ordered groups where appropriate, for categorical variables. A search for cut-off points to allow analysis of the cathepsin-D as a categorical variable was considered to be justified after it had been verified in univariate and multivariate tests for trend using Cox regression analysis (Cox, 1972) that higher levels of cathepsin-D were significantly associated with a poor (relapse-free) survival. For this search, isotonic regression analysis (IRA) with the length of relapse-free survival as end point (Barlow et al, 1972; Foekens et al, 1994), with modifications, was used. In the modified analysis, the reference value for the relative relapse rate was set at 1 at the median cathepsin-D concentration of 47 pmol $mg^{-1}$ protein. Moreover, IRA was performed after correction for age and menopausal status, tumour size, the number of positive lymph nodes, adjuvant therapy, ER and PgR. In addition, spline regression analysis (Gray, 1992) was performed to compare the fitted step function of the IRA with a smooth transformation from the spline regression analysis. Relapse-free and overall survival probabilities were calculated by the actuarial method of Kaplan and Meier (1958). To prevent unreasonable influence of cathepsin-D outliers in regression analyses, we replaced values above the 95th and below the 5th percentiles by these values which were 125.14 and 16.85 pmol $mg^{-1}$ protein respectively. Both uni- and multivariate analysis, including tests for interactions, were performed using the Cox proportional hazard model. The associated likelihood ratio test was used to test for differences between models with variables in- and excluded. In the multivariate analyses, the unknowns for ER/PgR status, and nodal status, were treated as separate groups to allow inclusion of all 2810 patients in the final models. All computations were carried out with the STATA statistical package, release 5.0 (STATA Corp., College Station, TX, USA). All *P*-values were two-sided and relate to all available data unless otherwise indicated.

### RESULTS

### Levels and associations

The median cathepsin-D level measured with IRMA in breast cancer cytosols was 47 pmol $mg^{-1}$ protein (range 0–902 pmol $mg^{-1}$ protein; mean ± s.d. 58 ± 48 pmol $mg^{-1}$ protein). Figure 1 shows the log-normal distribution of cathepsin-D levels in 2810 cytosols.

**Table 2** Cox univariate and multivariate analysis of relapse-free and overall survival

| Factor | Relapse-free survival | | | Overall survival | | |
|---|---|---|---|---|---|---|
| | Univariate *P*-value | Multivariate *P*-value | Relative relapse rate[a] | Univariate *P*-value | Multivariate *P*-value | Relative death rate[a] |
| Age and menopausal status | < 0.0001[b] | < 0.0001[b] | | < 0.0001[b] | < 0.0001[b] | |
| Age premenopausal[c] | | | 0.67 (0.59–0.76) | | | 0.74 (0.63–0.86) |
| Age post menopausal[c] | | | 0.92 (0.84–1.00) | | | 1.21 (1.11–1.33) |
| Post vs. premenopausal[d] | | | 1.44 (1.17–1.78) | | | 1.41 (1.09–1.82) |
| Tumour size | < 0.0001 | < 0.0001 | | < 0.0001 | < 0.0001 | |
| 2–5 cm vs ≤ 2 cm | | | 1.40 (1.23–1.59) | | | 1.43 (1.24–1.65) |
| > 5 cm vs ≤ 2 cm | | | 1.98 (1.63–2.39) | | | 1.96 (1.60–2.39) |
| Nodal status | < 0.0001 | < 0.0001 | | < 0.0001 | < 0.0001 | |
| N1–3 vs N0 | | | 1.99 (1.70–2.34) | | | 2.08 (1.75–2.47) |
| N>3 vs N0 | | | 3.70 (3.17–4.31) | | | 3.47 (2.94–4.10) |
| Adjuvant therapy (yes vs no) | < 0.0001[e] | < 0.0001 | 0.62 (0.53–0.72) | < 0.0001[e] | 0.002 | 0.78 (0.66–0.91) |
| ER/PgR status[f] | 0.004 | 0.12 | | < 0.0001 | < 0.0001 | |
| High/low vs low/low | | | 0.95 (0.77–1.16) | | | 0.81 (0.66–1.00) |
| Low/high vs low/low | | | 0.81 (0.61–1.08) | | | 0.65 (0.47–0.89) |
| High/high vs low/low | | | 0.85 (0.73–0.99) | | | 0.61 (0.52–0.71) |
| Cathepsin D status[g] | < 0.0001 | < 0.0001 | | < 0.0001 | < 0.0001 | |
| Q2 vs Q1 | | | 1.27 (1.08–1.50) | | | 1.36 (1.13–1.64) |
| Q3 vs Q1 | | | 1.45 (1.24–1.71) | | | 1.52 (1.26–1.82) |
| Q4 vs Q1 | | | 1.48 (1.26–1.74) | | | 1.56 (1.31–1.87) |

[a]Relative hazard rate (95% confidence interval). In the final multivariate models, all 2810 patients were included. [b]Age and menopausal status combined. [c]Age in decades tested separately for pre- and post-menopausal patients. [d]Post menopausal as compared with premenopausal. [e]Node-positive patients only. [f]High vs low: ≥ 10 vs < 10 fmol $mg^{-1}$ protein. [g]Q1: 0–33, Q2: > 33–47, Q3: > 47–70 and Q4: > 70 pmol $mg^{-1}$ protein respectively.

 © Cancer Research Campaign 1999

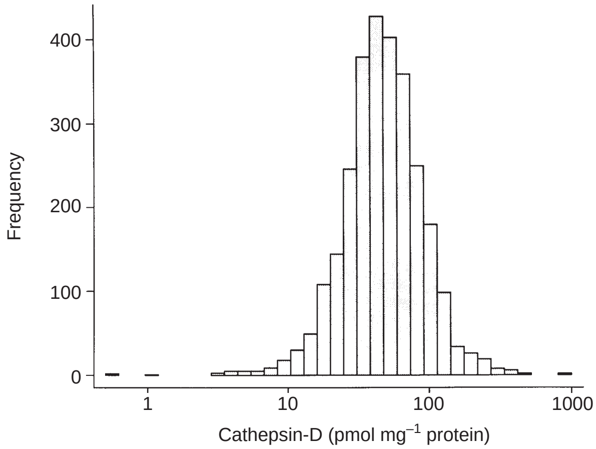

**Figure 1** Distribution of cathepsin-D over 2810 human primary breast tumour cytosols

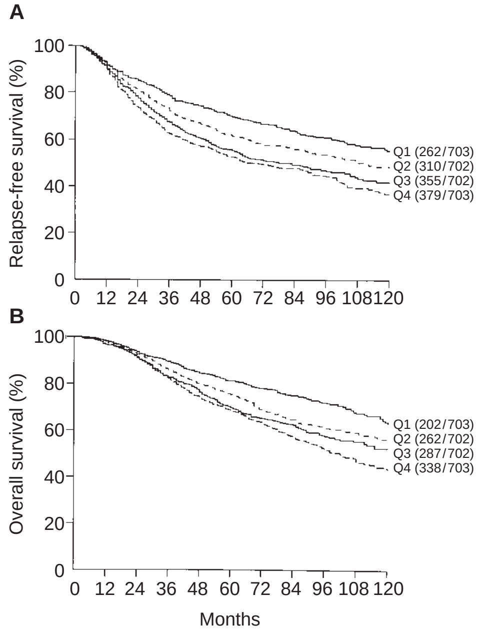

**Figure 2** Actuarial relapse-free (A) and overall survival (B) as a function of the level of cathepsin-D divided by quartiles. Q1: 0–33, Q2: > 33–47, Q3: > 47–70, and Q4: > 70 pmol mg$^{-1}$ protein respectively. Numbers between parentheses indicate the number of failures/total number of patients in each group

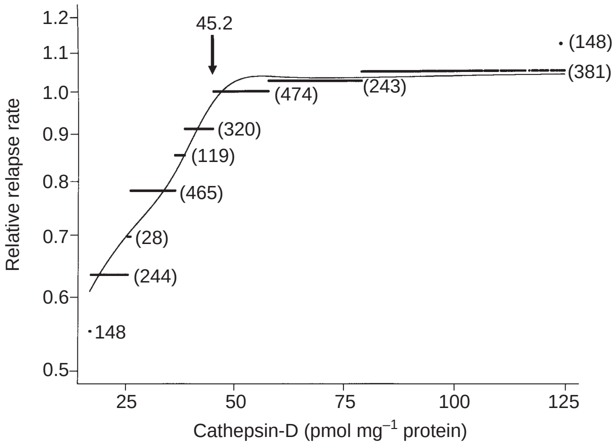

**Figure 3** Isotonic regression analysis for relapse-free survival as a function of cathepsin-D levels. Expressed are the relative relapse rates as a function of the level of cathepsin-D with the median of 47 pmol mg$^{-1}$ protein as reference value (1.0). Values are corrected for age/menopausal status, tumour size, the number of positive lymph nodes, adjuvant therapy, ER and PgR status. Step-function: isotonic regression analysis. Smooth curve: spline regression analysis. Cathepsin-D values were shrunk at the 5% and 95% percentiles, with the values below and above set at 16.85 and 125.14 respectively. At both extreme percentiles, the point estimates in the figure represent 148 patients. Numbers between parentheses indicate the number of patients grouped by isotonic regression analysis. Arrow indicates position of cut-off point at 45.2 pmol cathepsin-D mg$^{-1}$ protein

In Table 1, the percentage of tumours with cathepsin-D levels divided in quarters is shown in relation to patient and tumour characteristics. There was no significant relationship between cathepsin-D levels and grade of the tumour. Higher levels of cathepsin-D were found in tumours of older, post-menopausal and node-positive patients. Moreover, higher cathepsin-D levels were measured in larger and steroid hormone receptor-positive tumours (Table 1). The Spearman rank correlation coefficients between cathepsin-D levels and age ($r_s$ = 0.05), and the levels of ER ($r_s$ = 0.12) and PgR ($r_s$ = 0.11), were very weak albeit statistically significant because of the large number of samples.

### Univariate analysis of relapse-free and overall survival

In univariate Cox regression analysis using log-transformed cathepsin-D values, increasing levels of cathepsin-D were associated with poor relapse-free (chi-squared = 57, d.f. = 1, $P$ < 0.0001) and overall survival (chi-squared = 51, d.f. = 1, $P$ < 0.0001). Also, after dividing cathepsin-D levels in quarters, higher levels were associated with an early relapse (chi-squared = 53, d.f. = 3, $P$ < 0.0001) and death (chi-squared = 54, d.f. = 3, $P$ < 0.0001). The relative relapse rates (including their 95% confidence interval), set at 1 for tumours containing cathepsin-D levels ranging from 0 to 33 pmol mg$^{-1}$ protein (quarter 1: Q1), increased from 1.27 (1.07–1.49), via 1.55 (1.32–1.82) to 1.71 (1.46–2.00) for tumours containing cathepsin-D levels in the second (Q2), third (Q3) and fourth quarters (Q4) respectively. Similarly, compared with tumours containing cathepsin-D levels in Q1, the relative death rates of patients with cathepsin-D levels belonging to Q2, Q3 and Q4 increased from 1.39 (1.15–1.67), via 1.59 (1.33–1.90) to 1.87 (1.57–2.22). The Kaplan–Meier curves visualizing the 10-year relapse-free and overall survival probabilities as a function of cathepsin-D levels in quarters illustrate the increased rates of relapse and death with increasing levels of cathepsin-D (Figure 2). At 10 years, the difference in relapse-free survival probability between patients with the lowest 25% cathepsin-D levels (Q1: 55% ± 3% relapse-free) compared with the highest 25% cathepsin-D levels (Q4: 36% ± 3% relapse-free) was 18%. Similarly, at 10 years of follow-up, the difference in overall survival probabilities between the Q1 and Q4 groups was 20% (Q1: 63% ± 3%, and Q4: 43% ± 3% deaths respectively).

### Multivariate analysis of relapse-free and overall survival

Table 2 shows that the classical prognostic factors, age/ menopausal status, tumour size and the number of positive lymph

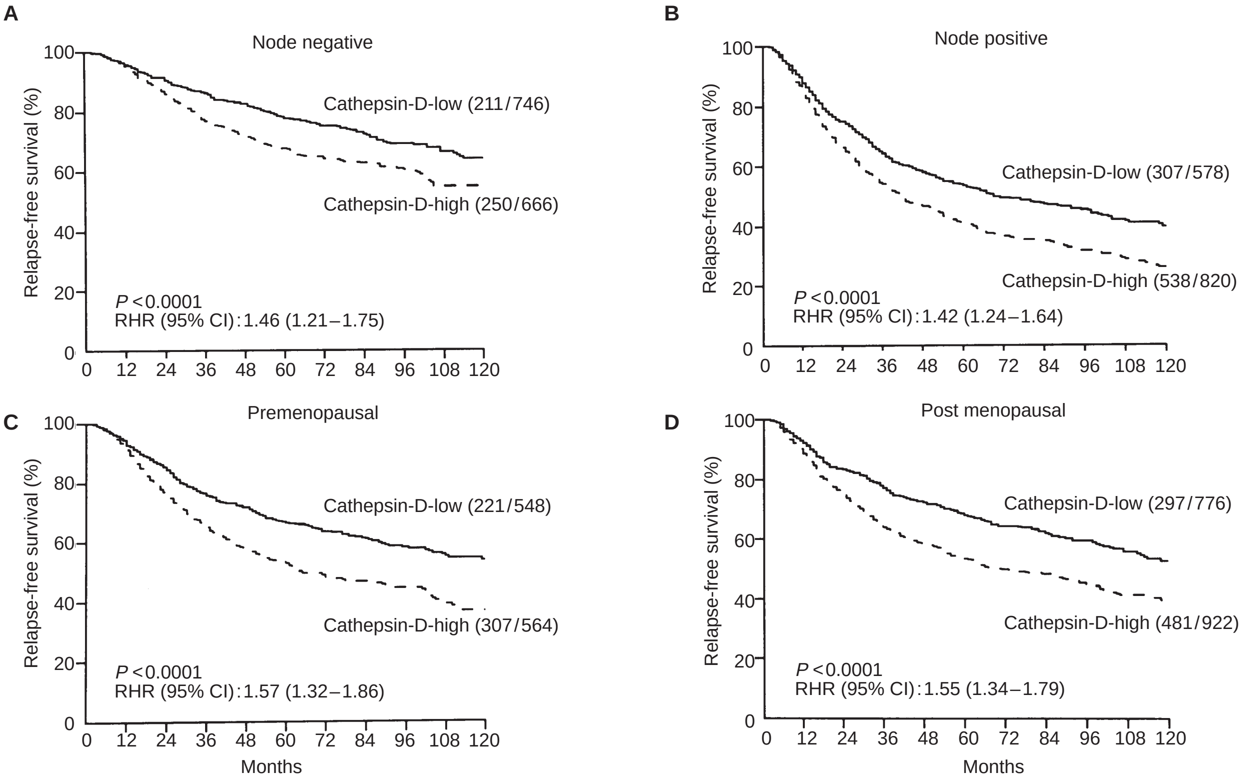

**Figure 4** Actuarial relapse-free survival as a function of cathepsin-D status in subgroups of node-negative (A), node-positive (B), premenopausal (C) and post-menopausal patients (D). Cathepsin-D-low: cathepsin-D levels < 45.2 pmol mg$^{-1}$ protein; cathepsin-D-high: cathepsin-D levels ≥ 45.2 pmol mg$^{-1}$ protein. Numbers between parentheses indicate the number of failures/total number of patients in each group. RHR (95% CI): relative hazard rate (95% confidence interval)

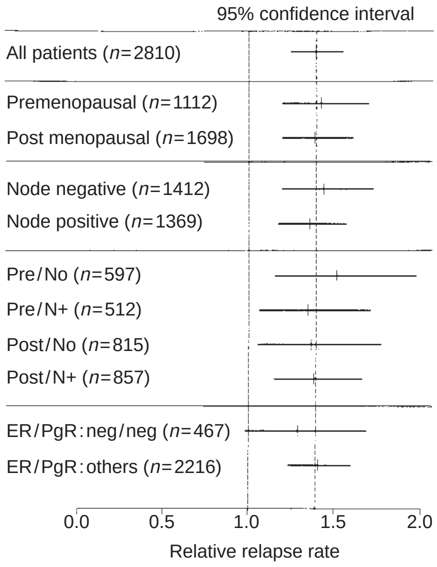

**Figure 5** Multivariate analysis for relapse-free survival in subgroups of patients as a function of cathepsin-D status. Data shown are point estimates with 95% confidence interval of patients with cathepsin-D tumour levels ≥ 45.2 pmol mg$^{-1}$ protein, compared with values < 45.2 pmol mg$^{-1}$ protein (set at 1.0), after correction for age/menopausal status, tumour size, nodal status, adjuvant therapy, and/or ER/PgR. Numbers between parentheses indicate the number of patients in each (sub)group. The dotted vertical line indicates a relative relapse rate of 1.39, which belongs to tumours with cathepsin-D values ≥ 45.2 pmol mg$^{-1}$ protein in all 2810 patients nodes, which were strong prognostic factors in univariate analysis, significantly added to the multivariate models for relapse-free and overall survival. Adjuvant therapy in node-positive patients was associated with a favourable prognosis in univariate analysis and, when included as an indicator variable, also significantly contributed to both the multivariate models. Steroid hormone receptor status, which was a significant predictor for a favourable relapse-free and overall survival in univariate analysis, was an independent factor in analysis for overall survival only. Grade of differentiation, which was associated with a poor prognosis in univariate analysis for both relapse-free and overall survival (both $P < 0.0001$), was omitted from the multivariate models because many values were missing.

The independent predictive effect of cathepsin-D on relapse-free and overall survival was assessed with Cox multivariate analysis including age/menopausal status, tumour size, the number of positive lymph nodes, adjuvant therapy and ER/PgR status. Corrected for the classical prognostic factors, when used as a categorical variable divided by quartiles, cathepsin-D significantly predicted an early relapse (increase in chi-squared: 29, d.f. = 3, $P < 0.0001$) and death (increase in chi-squared: 29, d.f. = 3, $P < 0.0001$) (Table 2). When cathepsin-D was added as a log-transformed continuous variable, instead of as a categorical variable to the multivariate models, similar increases in chi-squared were observed, confirming its strong independent association with a poor relapse-free (increase in chi-squared: 29, d.f. = 1, $P < 0.0001$) and overall survival (increase in chi-squared: 25, d.f. = 1, $P < 0.0001$). This similar increase in chi-squared with less degrees of freedom suggests that cathepsin-D should rather be considered as a

 © Cancer Research Campaign 1999

continuous variable instead of as a categorical variable (see also below). There were no statistically significant interactions between the classical prognostic factors, or between cathepsin-D and any of the classical prognostic factors, in analysis for relapse-free survival.

### Assessment of cut-off point for cathepsin-D

In the reported literature, cathepsin-D is almost exclusively analysed as a dichotomized variable (Westley and May, 1996; Ferrandina et al, 1997). To enable comparison of our data with those reported in the literature, and to classify tumours as cathepsin-D-high and -low, we have searched for an optimized cut-off point in our cohort of patients. We considered this search justified because in a test for trend logarithmically transformed cathepsin-D levels were (independently) associated with a poor prognosis (see above), and also when analysed as a categorical variable classified by quartiles (Figure 2, Table 3). For this search, we have employed isotonic regression analysis (Barlow et al, 1972; Foekens et al, 1994) with the rate of relapse as end point. The relative relapse rate belonging to the median cathepsin-D value of 47 pmol $mg^{-1}$ protein was set at 1. Corrected for age, menopausal status, tumour size, the number of positive lymph nodes, adjuvant therapy and ER/PgR status, the results from isotonic regression analysis showed a stepwise increase in the rate of relapse with cathepsin-D levels increasing from $\leq$ 16.9 to 45.2 pmol $mg^{-1}$ protein. At higher levels of cathepsin-D, the relative relapse rate was more or less constant (Figure 3). The results of the isotonic regression analysis match very well with those of cubic spline regression (Figure 3, solid line). In fact, there is no clear indication for a cut-off point because the analyses suggest a continuous increase of the relapse rate for cathepsin-D values up to $\pm$ 45 pmol $mg^{-1}$ protein. However, to allow comparison of our results with those of others, we have chosen a cathepsin-D level of 45.2 pmol $mg^{-1}$ protein as a cut-off point to define tumours as cathepsin-D-high and -low. Using this cut-off point, 53% of the tumours were classified as cathepsin-D-high and 47% as cathepsin-D-low.

### Subgroup analysis

In subsequent exploratory analyses, we have analysed the association of cathepsin-D, used as a dichotomized variable, with relapse-free survival in clinically relevant subgroups of patients. Figure 4 shows the 10-year relapse-free probability as a function of high and low cathepsin-D levels in subgroups of node-negative (Figure 4A), node-positive (Figure 4B), premenopausal (Figure 4C) and post-menopausal patients (Figure 4D). As expected from the observed lack of interaction between cathepsin-D and the various classical prognostic parameters in univariate analysis for relapse-free survival, the prognostic value of cathepsin-D was almost equally strong in the four subgroups of patients. The relative relapse rates for tumours with high cathepsin-D levels, compared with those with low cathepsin-D levels, ranged from 1.42 to 1.57 (Figure 4). This similar prognostic strength of cathepsin-D in the various subgroups of patients is further illustrated in Figure 5, showing comparable relative relapse rates as a function of cathepsin-D status in subgroups of patients, after correction for age/menopausal status, tumour size, nodal status and steroid hormone receptor status.

### DISCUSSION

In the literature, there is controversy regarding the prognostic significance of cathepsin-D in primary breast cancer. This dispute originates from studies using either immunohistochemistry or Western blotting techniques (Henry et al 1990; Tandon et al, 1990; Domagala et al, 1992; Isola et al, 1993; Ravdin, 1993; Ravdin et al, 1994; Cardiff, 1996; Rochefort, 1996). Henry et al (1990) reported that immunohistochemically assessed cathepsin-D in tumour cells was associated with a favourable prognosis in node-positive patients. In contrast, Isola et al (1993) in a study involving 262 node-negative patients showed that tumour cell-associated cathepsin-D expression was associated with a poor prognosis. These latter investigators furthermore showed that cathepsin-D expression in macrophages was not significantly related to prognosis. The opposite, i.e. expression of cathepsin-D by host cells rather than by the tumour cells is related with a poor prognosis, was reported in several other studies (Têtu et al, 1993; Joensuu et al, 1995; O'Donoghue et al, 1995; Nadji et al, 1996). However, in none of these latter studies was cathepsin-D an independent prognostic variable.

From many studies in which cathepsin-D status was assessed by ELISA or IRMA in tumour cytosols, like the present one, there is agreement between the results of the various studies. They virtually all show an adverse prognosis with increasing cathepsin-D levels, in many cases also in multivariate analysis (reviewed by Rochefort, 1994; Westley and May, 1996). It is, however, not surprising that different methods give different results. Measurement of cathepsin-D by IRMA in cytosols will result in an estimate of the cathepsin-D level originating from tumour cells and host cells. The advantage of immunohistochemistry is that the expression of cathepsin-D by the different cell types, such as tumour cells and host macrophages, can be studied separately. Various studies have been performed to address the relative contribution of the different cell types responsible for the cathepsin-D level in tumour cytosols. The cytosolic cathepsin-D level was shown to correlate well with cathepsin-D expression in cancer cells (Maudelonde et al, 1992; Remmele and Sauer-Manthey, 1993; Roger et al, 1994). This correlation was found to be stronger than that between the cytosolic cathepsin-D level and the number of macrophages in the tumour (Remmele and Sauer-Manthey, 1993; Roger et al, 1994). Also regarding this aspect, there is no agreement and the reverse has been reported (Razumovic et al, 1997). We fully agree with the statement which has been put forward previously by Rochefort (1996), 'one should not mix data obtained by well-standardized and controlled cytosolic assays with those obtained by immunohistochemistry using different antibodies without standardized quantification'.

As mentioned above, virtually all studies addressing the prognostic value of cytosolic cathepsin-D level with breast cancer prognosis show that a high level is associated with a poor relapse-free and overall survival (reviewed by Westley and May, 1996). However, there is no consensus with respect to its prognostic value in node-negative patients. In a recent meta-analysis involving 2690 node-negative patients, it was shown that a high level of cathepsin-D was associated with a poor relapse-free survival (Ferrandina et al, 1997). In the present study involving 1412 node-negative patients, cathepsin-D was also found to be significantly associated with a poor relapse-free survival, and also in multivariate analysis (Figure 5). Moreover, we observed no statistically significant interaction between cathepsin-D and any of the

© Cancer Research Campaign 1999

classical prognostic factors in analysis for relapse-free survival. Therefore, there are no reasons to assume that the prognostic value of cathepsin-D would be different for the various subgroups of patients. Indeed, the relative relapse rates for patients with high tumour levels of cathepsin-D, compared with those with low levels in the various clinically relevant subgroups of patients, were similar, and their 95% confidence intervals all showed an overlap (Figure 5).

In most studies in which cathepsin-D level was determined in cytosols, a lack of significant relationships between cathepsin-D and classical prognostic factors was reported. Also in the present study, the relationships of cathepsin-D with older age and post-menopausal status, larger tumour size, the number of positive lymph nodes, ER and PgR were weak but statistically significant because of the large numbers of patients (Table 1). These weak associations may, thus, have no clinical relevance. However, as has been discussed by Westley and May (1996), the most important question is not whether cathepsin-D relates to other prognostic factors, but whether cathepsin-D is a prognostic factor in its own right and is able to predict relapse-free and overall survival. From many studies (Westley and May, 1996), including the present large study and a recent meta-analysis (Ferrandina et al, 1996), it is clear that a high level of cathepsin-D, when measured by IRMA or ELISA in cytosolic extracts, is strongly associated with a poor prognosis in patients with primary breast cancer. The IRMA is convenient, can be quality controlled (Benraad et al, 1992), and can be performed on the same cytosols which are routinely prepared for ER and PgR estimations (EORTC Breast Cancer Cooperative Group, 1980).

### ACKNOWLEDGEMENTS

We are indebted to Henk Portengen and Erica Binnendijk-Noordegraaf for technical assistance, and we gratefully express our thanks to the surgeons, pathologists and internists of the St. Clara Hospital Rotterdam, Ikazia Hospital Rotterdam and St. Franciscus Gasthuis Rotterdam for the supply of tumour tissues and/or for assisting us in the collection of the clinical follow-up data. This study was supported by grant DDHK 96-1234 of the Dutch Cancer Society (NKB).

### REFERENCES

Barlow RE, Bartholomew DJ, Bremner JM and Brunk HD (1972) *Statistical Interference Under Order Restrictions*. John Wiley & Sons: London

Benraad ThJ, Geurts-Moespot A, Sala M, Piffanelli A, Ross A and Foekens JA on behalf of the EORTC Receptor Study Group (1992) Quality control of cathepsin D measurement by the EORTC Receptor Study Group. *Eur J Cancer* **28**: 72–75

Briozzo P, Morisset M, Capony F, Rougeot C and Rochefort H (1988) In vitro degradation of extracellular matrix with $M_r$ 52 000 cathepsin D secreted by breast cancer cells. *Cancer Res* **48**: 3688–3692

Briozzo P, Badet J, Capony F, Pieri I, Montcourrier P, Barritault D and Rochefort H (1991) MCF-7 mammary cancer cells respond to bFGF and internalize it following its release from extracellular matrix: a permissive role of cathepsin D. *Exp Cell Res* **194**: 252–259

Cardiff RD (1994) Cathepsin D and breast cancer: useful? *Hum Pathol* **25**: 847–848

Conover CA and De Leon DD (1994) Acid-activated insulin-like growth factor-binding protein-3 proteolysis in normal and transformed cells: role of cathepsin D. *J Biol Chem* **269**: 7076–7080

Cox DR (1972) Regression models and life tables. *J R Stat Soc* **34**: 187–220

Duffy MJ, O'Grady P, Devaney D, O'Sorian L, Fennelly JJ and Lijnen HJ (1988) Urokinase-plasminogen activator, a marker for aggressive breast cancers. *Cancer* **62**: 531–533

Domagala W, Striker G, Szadowska A, Dukowicz A, Weber K and Osborn M (1992) Cathepsin D in invasive ductal NOS breast carcinoma as defined by immunohistochemistry: no correlations with survival at 5 years. *Am J Pathol* **141**: 1003–1012

Emmert-Buck MR (1996) Cathepsin D and prognosis in breast cancer: one piece of a larger puzzle? *Hum Pathol* **27**: 869–871

EORTC Breast Cancer Cooperative Group (1980) Revision of the standards for the assessment of hormone receptors in human breast cancer. *Eur J Cancer* **16**: 1513–1515

Ferrandina G, Scambia G, Bardelli F, Panici PB and Massori A (1997) Relationship between cathepsin-D content and disease-free survival in node-negative breast cancer patients: a meta-analysis. *Br J Cancer* **76**: 661–666

Foekens JA, Portengen H, van Putten WLJ, Peters HA, Krijnen HLJM, Alexieva-Figusch J and Klijn JGM (1989) Prognostic value of estrogen and progesterone receptors measured by enzyme immunoassays in human breast tumor cytosols. *Cancer Res* **49**: 5823–5828

Foekens JA, van Putten WLJ, Portengen H, de Koning HYWCM, Thirion B, Alexieva-Figusch J and Klijn JGM (1993) Prognostic value of PS2 and cathepsin D in 710 human primary breast tumors: multivariate analysis. *J Clin Oncol* **11**: 899–908

Foekens JA, Schmitt M, van Putten WLJ, Peters HA, Portengen H, Kramer MD, Jänicke F and Klijn JGM (1994) Plasminogen activator inhibitor-1 and prognosis in primary breast cancer. *J Clin Oncol* **12**: 1648–1658

Foekens JA, Berns EMJJ, Look MP and Klijn JGM (1996) Prognostic factors in node-negative breast cancer (1996). In *Hormone-Dependent Cancer*. Pasqualini JR and Katzenellebogen BS (eds), pp. 217–252. Marcel Dekker: NY

Garcia M, Derocq D, Pujol P and Rochefort H (1990) Overexpression of transfected cathepsin D in transformed cells increases their malignant phenotype and metastatic potency. *Oncogene* **5**: 1809–1814

Gray RJ (1992) Flexible methods for analyzing survival data using splines, with applications to breast cancer prognosis. *J Am Stat Assoc* **87**: 942–952

Henry JA, McCarthy AL, Angus B, Westley BR, May FEB, Nicholson S, Cairns J, Harris AL and Horne CHW (1990) Prognostic significance of the estrogen-regulated protein, cathepsin D, in breast cancer. *Cancer* **65**: 265–271

Isola J, Weitz S, Visakorpi T, Holli K, Shea R, Khabbaz N and Kallioniemi OP (1993) Cathepsin D expression detected by immunohistochemistry has independent prognostic value in axillary node-negative breast cancer. *J Clin Oncol* **11**: 36–43

Jänicke F, Schmitt M, Hafter R, Hollreider A, Babic R, Ulm K, Grössner W and Graeff H (1990) Urokinase-type plasminogen activator (u-PA) antigen is a predictor of early relapse in breast cancer. *Fibrinolysis* **4**: 69–78

Joensuu H, Toikkanen S and Isola J (1995) Stromal cell cathepsin D expression and long-term survival in breast cancer. *Br J Cancer* **71**: 155–159

Kaplan EL and Meier P (1958) Nonparametric estimation from incomplete observations. *J Am Stat Assoc* **53**: 457–481

Kute TE, Shao ZM, Sugg NK, Long RT, Russell GB and Case LD (1992) Cathepsin D as a prognostic indicator for node-negative breast cancer patients using both immunoassays and enzymatic assays. *Cancer Res* **52**: 5198–5203

Liaudet E, Derocq D, Rochefort H and Garcia M (1995) Transfected cathepsin D stimulates high density cancer cell growth by inactivating secreted growth inhibitors. *Cell Growth Differ* **6**: 1045–1052

Nadji M, Fresno M, Nassiri M, Conner G, Herrero A and Morales AR (1996) Cathepsin D in host stromal cells, but not in tumor cells, is associated with aggressive behavior in node-negative breast cancer. *Hum Pathol* **27**: 890–895

O'Donoghue AEMA, Poller DN, Bell JA, Galea MH, Elston CW, Blamey RW and Ellis IO (1995) Cathepsin D in primary breast carcinoma: adverse prognosis is associated with expression in cathepsin D in stromal cells. *Breast Cancer Res Treatment* **33**: 137–145

Ravdin P (1993) Evaluation of cathepsin D as a prognostic factor in breast cancer. *Breast Cancer Res Treatment* **24**: 219–226

Ravdin PM, Tandon AK, Allred CDC, Clark GM, Fuqua SAW, Hilsenbeck SH, Chamness GC and Osborne CK (1994) Cathepsin D by Western blotting and immunohistochemistry: failure to confirm correlations with prognosis in node-negative breast cancer. *J Clin Oncol* **12**: 467–474

Razumovic JJ, Stojkovic RR, Petrovecki M and Gamulin S (1997) Correlation of two methods for determination of cathepsin D in breast carcinoma (immunohistochemistry and ELISA in cytosol). *Breast Cancer Res Treatment* **43**: 117–122

Remmele W and Sauer-Manthey J (1993) Comparative biochemical and immunohistochemical studies on the cathepsin D content of human breast cancer. *Virchows Arch A Pathol Anat* **422**: 467–473

Rochefort H (1994) Oestrogens, proteases and breast cancer. From cell lines to clinical applications. *Eur J Cancer* **10**: 1583–1586

 *© Cancer Research Campaign 1999*

Rochefort H (1996) The prognostic value of cathepsin D in breast cancer. A long road to the clinic. *Eur J Cancer* **32A**: 7–8

Roger P, Montcourrier P, Maudelonde T, Brouillet J-P, Pages A, Laffarque F and Rochefort H (1994) Cathepsin D immunostaining in paraffin-embedded breast cancer cells and macrophages: correlation with cytosolic assay. *Hum Pathol* **25**: 863–871

Saftig P, Hetman M, Schmahi L, Weber K, Heine L, Mossman H, Koster A, Hess B, Evers M, von Figura K and Peters C (1995) Mice deficient for the lysosomal proteinase cathepsin D exhibit progressive atrophy of the intestinal mucosa and profound destruction of lymphoid cells. *EMBO J* **14**: 3599–3608

Spyratos F, Brouillet JP, Defrenne A, Hacène K, Rouesse J, Moudelonde T, Brunet M, Andrieu C, Desplaces A and Rochefort H (1989) Cathepsin-D: an independent prognostic factor for metastasis of breast cancer. *Lancet* **i**: 1115–1118

Tandon AK, Clark GM, Chamness GC, Chirgwin JM and McGuire WL (1990) Cathepsin D and prognosis in breast cancer. *N Engl J Med* **332**: 297–302

Têtu B, Brisson J, Côté C, Brisson S, Potvin D and Roberge N (1993) Prognostic significance of cathepsin D expression in node-positive breast carcinoma: an immunohistochemical study. *Int J Cancer* **55**: 429–435

Thorpe SM, Rochefort H, Garcia M, Freiss G, Christensen IJ, Khalaf S, Paolucci F, Pau B, Rasmussen BB and Rose C (1989) Association between high concentrations of $M_r$ 52 000 cathepsin D and poor prognosis in primary human breast cancer. *Cancer Res* **49**: 6008–6014

Vignon F, Capony F, Chambon M, Freiss G, Garcia M and Rochefort H (1986) Autocrine growth stimulation of the MCF-7 breast cancer cells by the estrogen-regulated 52 k protein. *Endocrinology* **118**: 1537–1540

Westley BR and Rochefort H (1979) Estradiol induced proteins in the MCF-7 human breast cancer cell line. *Biochem Biophys Res Commun* **90**: 410–416

Westley BR and Rochefort H (1980) A secreted glycoprotein induced by estrogen in human breast cancer cell lines. *Cell* **20**: 353–362

Westley BR and May FEB (1996) Cathepsin D and breast cancer. *Eur J Cancer* **32A**: 15–24

© Cancer Research Campaign 1999
